# Development and piloting of the Fiji Injury Surveillance in Hospitals System (TRIP Project-1)

**DOI:** 10.1016/j.injury.2011.10.007

**Published:** 2013-01

**Authors:** I. Wainiqolo, B. Kafoa, E. McCaig, B. Kool, R. McIntyre, S. Ameratunga

**Affiliations:** a College of Medicine, Nursing and Health Science, Fiji National University, Suva, Fiji; b Section of Epidemiology and Biostatistics, School of Population Health, The University of Auckland; Auckland, New Zealand

**Keywords:** Injury surveillance system, Injury prevention, Developing countries, Injury epidemiology

## Abstract

**Introduction:**

Whilst more than 90% of injury related deaths are estimated to occur in low-and-middle-income countries (LMICs), the epidemiology of fatal and hospitalised injuries in Pacific Island Countries has received scant attention. This study describes the development and piloting of a population-based trauma registry in Fiji to address this gap in knowledge.

**Methods:**

The Fiji Injury Surveillance in Hospitals (FISH) system was an active surveillance system designed to identify injuries resulting in death or a hospital admission in Viti Levu, Fiji. During the pilot conducted over five months in 2005, Accident and Emergency registers, admission folders and morgue registers from 8 of Viti Levu's 12 hospitals, and an additional 3 hospitals in other parts of the country were reviewed by hospital staff and medical students to identify cases and extract a minimum data set that included demographic factors; the mechanism, nature and context of injury; substance use; and discharge outcomes. The system was audited to identify and redress difficulties with data quality in a manner that also supported local capacity development and training in injury surveillance and data management.

**Results:**

This pilot study demonstrated the potential to collect high quality data on injuries that can pose a significant threat to life in Fiji using a mechanism that also increased the capability of health professionals to recognise the significance of injury as a public health issue.

**Conclusion:**

The injury surveillance system piloted provides the opportunity to inform national injury control strategies in Fiji and increase the capacity for injury prevention and more focused research addressing risk factors in the local context.

## Introduction

Injury accounts for 9% of global mortality and represents up to 12% of the global burden of disease.^1^ Whilst more than 90% of injury related deaths are estimated to occur in low-and-middle-income countries (LMIC),^1,2^ policy investment to address the causes and consequences of injury in these settings remains grossly inadequate. One underlying reason for this is the relative lack of local research evidence.^3^ This is an important public health issue in the small island nations in the South Pacific where despite a significant impact on scarce healthcare dollars, population-based data on injuries are largely limited to selected lists of health statistics.A review of the Fiji Health Annual Reports from 1988 to 2000 revealed that injury and poisoning were amongst the top five causes of deaths and hospitalisations in the country.^4–9^ Extrapolating from the World Health Organisation estimates regarding the economic impact of road traffic injuries alone,^1^ the costs imposed could approximate 15% of the total Fiji health budget. Yet, available data are inadequate to inform a robust national injury prevention strategy based on the causes and nature of injuries, as well as the high-risk groups involved. A systematic approach to monitor and address the burden of injuries in developing nations is long overdue in Pacific Island countries and elsewhere.^10–12^

In order to obtain a profile of the leading causes of death and hospitalisation due to injury in Fiji, a project specific data collection system – the Fiji Injury Surveillance in Hospitals (FISH) – was established as a component of the Traffic Related Injury in the Pacific (TRIP) project. The latter comprises a collaborative research initiative involving the Fiji School of Medicine, The Fiji Ministry of Health and the University of Auckland, funded by The Wellcome Trust and the Health Research Council of New Zealand. The overall goals of the FISH system were to characterise the burden of fatal and hospitalised injury in Fiji with regard to the major causes of life-threatening injuries and high risk populations involved, and to establish the foundation for national injury prevention and control strategies. This paper describes the development and piloting of the FISH system, and highlights some of the issues encountered.

## Methods

### Surveillance setting

The Republic of Fiji comprises an archipelago of over 300 islands with a total population of about 840,000 at the 2007 Census.^13^ The overall aim of the project was to obtain a population-based profile of fatal and hospitalised injuries in Viti Levu, the largest island of Fiji with 70% of the national population. For the purposes of the 5-month pilot phase (1 January 2005 to 30 April 2005), the proposed active injury surveillance system was established in 8 out of the 12 trauma admitting hospitals in Viti Levu. Although not directly relevant to the final surveillance system, the pilot also included 3 hospitals in other parts of the country.

### Development

The WHO injury surveillance guidelines (2001) were used to develop and implement the FISH system.^2^ The objectives for this phase of the project were to: (1) develop and pre-test the proposed data collection process and study instrument; (2) assess the feasibility of recruitment, its success and barriers and modify the research protocol accordingly; and (3) implement the revised system.

In the early stages of development, the researchers held meetings with health personnel at national, divisional and sub-divisional levels of the Fiji Ministry of Health, and other key injury stakeholders. The objectives of these meetings were to: (1) raise awareness of the injury research being undertaken; (2) seek approval to undertake injury surveillance in the study hospitals; and (3) conduct a needs assessment to identify potential injury data sources and assess available resources that the TRIP team could utilise.

Given the constrained resources within health services, the project team sought opportunities to utilise existing health information where relevant, avoid duplication of processes, and develop the capacity and capability of local staff and students.

Ethical approval for the study was obtained from the Fiji National Research Ethics Review Committee of the Ministry of Health.

#### Case inclusion criteria and identification

The FISH system was designed to capture data on all injuries that resulted in a death or a hospital admission for more than 12 h. Deaths prior to hospital admission were identified through the mortuaries that investigate all injury-related fatalities in Fiji. The WHO definition of *injury* was applied referring to “physical damage that results when a human body is suddenly or briefly subjected to intolerable levels of energy.”^2^

**Fig. 1:**
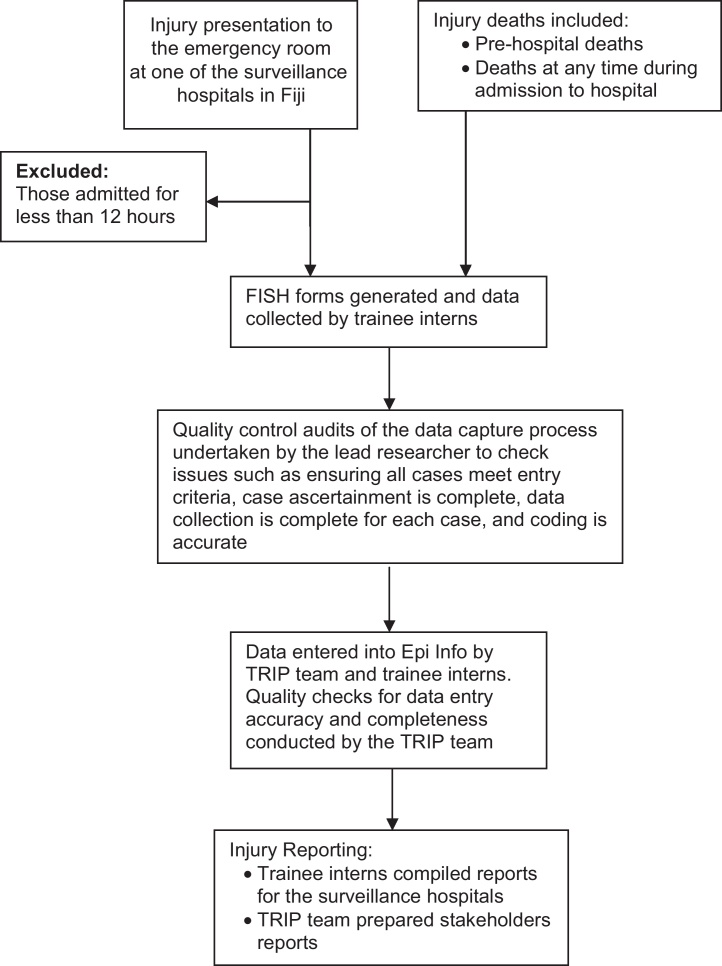
Fiji Injury Surveillance in Hospitals System: data collection process.

In order to increase the efficiency of resource utilisation and develop local capacity for public health surveillance, case identification was undertaken by final year medical students (trainee interns) on their community health rotations alongside nursing staff at each surveillance hospital (Fig. 1). These individuals systematically scanned accident and emergency registers, admission folders and morgue registers from the surveillance hospitals on a weekly or fortnightly basis to identify cases meeting the case inclusion criteria.

#### The injury minimum data set

Using the WHO injury surveillance guidelines injury form as a template,^2^ project staff developed a one page 23-item form that captured demographic data (name, age, gender, ethnicity); details regarding the injury event (place, activity, mechanism, intent, nature of injury, use of alcohol, kava and other substances); and the hospitalisation (date and time of admission and discharge, use of intensive care facilities and outcomes (Table 1). The nature and mechanisms of injury were classified using broad categories generally aligned to the principles of the International Classification of Diseases (ICD) Version 10 coding and the International Classification of the External Causes of Injury (ICECI).^14^ The use of kava (a drink from the root of *Piper methysticum*) was captured as this popular traditional intoxicant is widely consumed as a recreational beverage by all sectors of Fiji's society.^15^ A data dictionary and coding manual were developed by the TRIP team (FISH guideline) to provide information on the data collection processes for the study, and included variable definitions for the injury surveillance form.

#### Data collection and management

Using the standardised surveillance form, data collectors at each hospital abstracted the required data from patient records and entered the information into the study database (created in Epi Info Version 6).^16^

Four training workshops on all aspects relating to data collection, data entry and chart reviews were conducted for the trainee interns who had primary responsibility for these tasks at the surveillance hospitals. Nurses and pathologists also attended these training workshops as they would work alongside the trainee interns once the pilot phase was completed to undertake data collection for the remaining 7 months of the project. Most of the trainee interns involved at pilot sites had not previously participated in collaborative research and were not familiar with using statistical software. The training focused on raising awareness of the burden of injury for both the global and local contexts; orientation to the injury surveillance form and its accompanying guidelines; issues of patient confidentiality and privacy; interviewing techniques; and familiarisation with Epi Info the database software.

The TRIP research team was responsible for coordinating the data collection processes at all hospitals engaged in the pilot. In order to examine data quality and the integrity of the process, one of the Pacific research managers systematically audited all injury surveillance forms on at least a monthly basis cross checking FISH data with hospital registers and case notes. This was to ensure that cases met the inclusion criteria, case ascertainment was complete, and coding was accurate. Quality control checks of the database at all surveillance sites were undertaken by the lead researchers to ensure data entries were accurate and complete. Through interactive sessions during the pilot phase, feedback was gathered from the trainee interns involved in data collection regarding the surveillance process, including any ambiguities or difficulties.

## Results

### Process review

During the 5-month pilot phase, 183 forms for injury cases were completed. During the data quality audits, one duplicate entry and nine missing forms were identified, giving a case ascertainment rate of 95.3% (182/191). Missing data for individual items (recorded as ‘unknown’ or ‘unavailable’) were noted in 173 forms, most commonly for the following variables: date and time of injury, hospital number, and alcohol and substance use (Table 2). Potential explanations for the high levels of missing data in the substance abuse fields included poor recall by cases of the injury including substance use, and a degree of stigma attached to alcohol and kava use which may have increased the tendency to deny usage.

Research staff identified several difficulties that required particular attention during the pilot phase. These included clarity around case eligibility, and uncertainty with classifications such as intent, place of injury, and mechanism of injury. Examples of the decisions made by the research team regarding these issues are provided in Table 3. Acknowledging the importance of ensuring the data collected was relevant for the context whilst amenable to analyses that would inform injury control, the project team sought to make adaptations that were consistent with standard injury surveillance definitions. In exceptional situations where non-standard data were gathered (e.g., ciguatera poisoning from ingesting affected fish), information was captured in a manner that enabled analyses with or without these cases.

Amendments made to the injury surveillance form included the addition of new variables considered important in the local health care and public policy context (e.g., ‘mode of admission’, ‘name of location where injury took place’), and the addition of specific coded response options for some variables (e.g., ‘in a conflict situation’ as an ‘activity’ category).

The surveillance protocol was amended to reflect the revisions to definitions and coding clarifications, and the FISH recording guideline was printed in a booklet forms and distributed to the medical students and nurses involved in data collection. The regular audits and site visits were used by TRIP researchers to provide on-going training in key aspects of injury surveillance and Epi Info software/analysis for all those involved in data collection and dissemination of pilot study findings.

Whilst not intended to be representative of the study sites, data collectors were supported in undertaking preliminary analyses of the injury data by the TRIP team. The findings (as an example of the opportunity afforded by the proposed registry) were presented and discussed with stakeholders at meetings convened by the divisional and sub-divisional hospitals, the Ministry of Health Non-Communicable Disease committee, the Accident and Injury sub-committee (AIS) and Fiji Medical Association. In addition as part of the Community Medicine training requirement for the Medical Programme, the trainee interns involved in the FISH project prepared a report summarising the key findings based on the data collected from the hospital they were attached to.

## Discussion

This pilot study has demonstrated the feasibility of capturing the burden of fatal and hospitalised injury in the resource-constrained context of Fiji. Building local workforce capacity in injury surveillance was central to the success of the pilot. The training sessions and site visits conducted by the TRIP team provided support the surveillance hospitals and ensured data quality standards were maintained.

The strengths of the study include the development and implementation of a context-specific active surveillance system in a setting where population-based epidemiologic data on injury is sparse. The rigour of the data being collected is assisted by the adaptation of international guidelines, using data collectors with health or clinical backgrounds, implementing an active surveillance system with audit and feedback loops to enhance the accuracy, completeness and quality of data. The full-scale system is designed to enable the calculation of injury incidence using population-based denominators, and monitor trends over time and across health service regions. Furthermore, aligning the FISH data collection categories to international guidelines also permit meaningful international comparisons in the future.

Stakeholder engagement played a central role in piloting the proposed system. The Ministry of Health was instrumental in engaging the Fiji-based TRIP research investigators in their Accident and Injury sub-committee (AIS). This engagement was a key factor in obtaining buy-in from the clinicians. The investigators were expected to not only chair the AIS meetings but to also keep the Committee and the Non-Communicable Diseases Taskforce informed of the progress and outcomes of the FISH project. Involvement with the AIS committee provided the opportunity to network with the Land Transport Authority, the Police Force, the National Road Safety Council, the Red Cross, the Ministry of Labour, the Ministry of Youth & Sport and other relevant agencies. The establishment of these networks assisted the research team with both the obtaining of data and the dissemination of findings.

The pilot study has highlighted the importance of pre-testing the data capture form. The advantages included enabling the research team to: (1) see the appropriateness and possible complexities of the proposed instrument; and (2) to have a clear understanding of the logistics and processes around the data collection-related activities. The involvement of medical students in the project enabled these future health professionals to gain a critical understanding of health information systems and the burden of injury in Fiji.

Limitations of the pilot include the restricted specificity of the injury categories resulting in the high counts in the response category termed “other”. Not surprisingly, the data form trialled inevitably lacks the level of detailed data captured in surveillance systems in many high-income countries with considerably greater resources. This is particularly apparent with regard to domains such as ‘comorbidity’ and ‘injury severity’. There is a great need for resource-efficient approaches to collecting data that could assist adjustment for case-mix in LMIC settings, particularly when considering trauma outcomes. The lack of socio-economic indicators in the data collection system also limits the opportunity to investigate the influence of broader social determinants.^17^ The study was limited to those admitted to hospital or who died and therefore represents the spectrum of injury where threat to life is more likely (with the exception of some categories such as near drowning). However, such a system will not necessarily capture injuries that may pose a significant threat to disability and longer-term psychosocial sequelae. The system is also inevitably biased by factors that may influence access to hospital-based health care. Whilst the specific characteristics that may underlie such biases in the Fiji context are not clear, it is likely that people living in remote or rural locations and those seeking alternative treatment providers such as traditional healers will be amongst those who are less well represented in this database. Given the absence of a national trauma registry or routinely collected national injury data at this level of detail, we were unable to investigate these potential biases in more detail.

Lyons et al. affirmed that there is no such thing as a perfect information system, highlighting the need to focus on whether the data supports the purpose of collection rather than imperfections of assessment *per se*.^18^ Determining an appropriate balance between sufficient detail and simplicity of the data collection process is always a challenge for surveillance systems. Notwithstanding the acknowledged limitations, the FISH system is designed to capture injury-related deaths, regardless of whether these occur before or during a hospital admission, an attribute that is uncommon in most trauma registries in high-income settings. The adoption of complementary approaches to hospital injury surveillance systems such as community-based surveys should be given consideration. The WHO STEPS Violence and Injury Module,^19^ UNICEF Child injury surveys, and national disability surveys are some examples of these approaches.

The process undertaken to develop and pilot the injury surveillance system in Fiji is consistent with similar hospital-based surveillance activities that have taken place in other LMIC.^3,20–22^ Hyder et al. describe a pilot study of a childhood injury emergency department surveillance system established in four cities in developing countries. Data was collected over a three to four month period and obtained from caregiver-completed questionnaires (context of injuries, risk factor information, use of safety measures), and clinical care information (injury severity, injury outcomes, costs of care estimates).^3^ Capacity development was identified as a critical success factor of the pilot, particularly including the provision of technical assistance for the database management during regular site visits by the research team. The establishment of a provincial injury surveillance system in five hospitals in Thailand adopted a trauma registry based approach.^21^ Although the surveillance process was supervised by staff at the epidemiology division of Ministry of Public Health, the authors noted the quality of data varied by hospital, and they identified inadequate human resourcing as a contributing factor to this variation. Tercero et al. describe a one-year ED based surveillance system established in Nicaragua.^22^ The completion of external cause codes was the main challenge facing the research team, in 20.3% of records this information was missing. Efforts to increase the completion of this field included raising awareness of the importance of cause of injury classification and e-code training. Ward et al. described the process of establishing a national injury surveillance system in accident and emergency departments in Jamaica.^20^ Many of the characteristics of the system were similar to the process trialled in Fiji, but these authors noted that integrating the data collection form into the patient registration and permanent medical record was key to ensuring completeness of data collection. This aspect requires greater attention in Fiji.

The FISH system was established as a component of a research project to obtain a full year's worth of data on fatal and hospitalised injury. As such this data system was not designed to collect data indefinitely. Rather, FISH was designed to identify leading causes of potentially life-threatening injuries and related demographic and contextual factors in Fiji in a manner that could both inform national injury prevention priorities and identify information that could enhance routinely collected data should a surveillance system be established in the future.

Further to this project and several other initiatives, there have been many developments relating to health information systems in Fiji. Current activities designed to improve the quality of routinely collected injury relevant information include: upgrading the patient information system used in hospitals, moving towards incorporating a national health index number, and standardising the recording of information collected on death certificates. More directly related to this pilot, the information collected during the 12-month active surveillance period of FISH itself enabled the description of the epidemiology of fatal and hospitalised injuries with in-depth analyses focusing on many related issues including burns, childhood injuries, head injuries, poisonings, and falls.

Subsequent to the conduct of the present study, Mitchell et al. published an evaluation framework for injury surveillance systems.^23^ The framework consists of 18 characteristics that assess three areas of an injury surveillance system: data quality, operational, and practical considerations. These criteria require specific consideration in future refinements of injury surveillance systems in the Pacific. In addition, there are imperatives to consider key areas requiring capacity development and research investment to reduce the burden of injuries in low- and middle-income countries, as identified by Chandran et al., specifically: improving the collection of injury data, defining the epidemiology of injuries, estimating the costs of injuries, understanding public perceptions regarding injury causation, and engaging with policy makers to improve injury prevention and control.^24^ The current study signals an important step in the process of addressing these needs in Pacific Island nations.

## Conflicts of interest

No conflict of interest exists between any of the authors and this research.

## Role of the funding source

This study was funded by an international collaborative research grant from the Wellcome Trust (UK) and the New Zealand Health Research Council. The funders had no involvement in the study design; collection, analysis and interpretation of the study data; the writing of the manuscript; or the decision to submit the manuscript for publication.

## Figures and Tables

**Table 1:** Fiji Injury Surveillance in Hospitals System, data elements in the injury surveillance form.

Variable name and description	Response categories		
Hospital name	Free text		
Case number	Hospital number/PATIs number/researcher generated unique identifier		
Date of birth	Numeric		
Date of injury	Numeric		
Date of admission & Date of discharge	Numeric		
Time of injury	Numeric		
Mode of admission	Within hospital	Transfer from…	Other
Place of injury	Free text		
Geo code (of where injury took place)	Numeric		
Home address	Free text		
Gender	Male	Female	Unknown
Ethnicity	Fijian	Indian	Other
	Unknown		
Place where injury occurred	Private house	School	
	Highway/street/road	Recreational area	
	Workplace	Other	Unknown
Activity (at time of injury)	Work	Leisure/play	
	Organised sport	Travel (not work related)	
	In a conflict situation	Other	Unknown
Mechanism/cause	Road traffic injury	Sexual assault	Stab/cut
	Fall	Fire/heat/electricity	Hit by person/object
	Choking/hanging	Drowning/near drowning	
	Poisoning	Unknown	Other
Intent	Unintentional	Intentional (self harm)	
	Intentional (assault/violence)		Undetermined
	Other	Unknown	
Nature of injury	Fracture	Sprain/strain	
	Cut/bite/open wound	Bruise	
	Burn	Head injury/concussion	
	Internal injury of chest/abdomen		
	Asphyxia	Other	Unknown
Alcohol use	Suspected	No	Unknown
Kava use	Suspected	No	Unknown
Other substances use	Suspected	No	Unknown
Injury severity	Minor	Moderate	Severe
Outcome of admission	Transfer	Discharged	Died whilst admitted
	Dead on arrival/died before admitted	Unknown	Other

**Table 2:** Fiji Injury Surveillance in Hospitals System: missing data in the injury surveillance form, *n* = 173.

Variable	Number of surveillance forms with data recorded as “unknown” or “not available”
	*n* (%)
Date of injury	15 (8.7)
Hospital number	50 (28.9)
Time of injury	67 (38.7)
Alcohol use^a^	51 (29.5)
Kava use^a^	62 (35.8)
Other substance use^a^	64 (36.9)

a Amongst records of cases aged ≥10 years.

**Table 3:** Fiji Injury Surveillance in Hospitals System: examples of classification difficulties encountered and addressed during the pilot.

Variable field	Example	Resolution	Consistent with ICD
Activity at time of injury	‘A carrier driver who has an accident, should his activity be work or travelling?’	Classified as work	Yes
Mechanism of injury	‘Does fish poisoning count as an injury?’	Decided to include provided they were caused by ciguatera or metals as these are an issue for the Pacific	No
Intent	‘Would animal bites be classed as intentional?’	Unintentional	Yes
Nature of injury	Large proportions of drowning and hanging recorded that did not fit into available coded categories	The category ‘asphyxia’ was created to address the nature of injury for these two mechanisms	No
